# Comparison of postoperative nausea and vomiting between Remimazolam and Propofol in Patients undergoing oral and maxillofacial surgery: a prospective Randomized Controlled Trial

**DOI:** 10.1186/s12871-023-02091-3

**Published:** 2023-04-22

**Authors:** Eun-Jung Kim, Cheul-Hong Kim, Ji-Young Yoon, Gyeong-Jo Byeon, Hee Young Kim, Eun-Ji Choi

**Affiliations:** 1 Department of Dental Anesthesia and Pain Medicine, School of Dentistry, Dental Research Institute, Pusan National University, Yangsan, Republic of Korea; 2 Dental and Life Science Institute, Pusan National University, Yangsan, Republic of Korea; 3 Department of Anesthesia and Pain Medicine, Pusan National University Yangsan Hospital, Yangsan, Republic of Korea; 4 Department of Anesthesia and Pain Medicine, School of Medicine, Pusan National University, Yangsan, Republic of Korea

**Keywords:** Postoperative nausea and vomiting, Propofol, Remimazolam

## Abstract

**Background:**

Remimazolam is a recently approved, ultra-short-acting benzodiazepine. However, few studies have investigated remimazolam in relation to postoperative nausea and vomiting (PONV). This study aimed to compare the effects of remimazolam and propofol on PONV in patients undergoing oral and maxillofacial surgery.

**Methods:**

Patients (n = 206) aged 19–65 years who were scheduled for oral and maxillofacial surgery were randomized into two groups, the remimazolam (R) and propofol group (P). In the R group (n = 94), remimazolam was used to induce anesthesia at 12 mg/kg/h and to maintain anesthesia at 1–2 mg/kg/h. In the P group (n = 95), anesthesia was induced and maintained with propofol (target effect-site concentration: 3–5 µg/ml). In both groups, remifentanil was administered at a target effect-site concentration of 2.5-4 ng/ml. The primary outcome was the overall incidence of PONV during the first 24 h after surgery. Secondary outcomes included the severity of nausea, use of rescue antiemetics, severity of postoperative pain, use of rescue analgesia, and quality of recovery.

**Results:**

The incidence of PONV during the first 24 h after surgery was 11.7% and 10.5% in the R group and P group, respectively, and there was no significant difference in the severity of nausea (P > 0.05). Ten patients in the R group and ten patients in the P group required rescue antiemetics during the first 24 h after surgery (P = 0.98). No inter-group differences were observed in terms of postoperative pain score, use of rescue analgesia, and quality of recovery (P > 0.05).

**Conclusions:**

In this study, remimazolam did not increase the incidence and severity of PONV compared with propofol.

**Trial registration:**

KCT0006965, Clinical Research Information Service (CRIS), Republic of Korea. Registration date: 26/01/2022.

## Background

Postoperative nausea and vomiting (PONV) is a distressing and unpleasant post-surgical complication. PONV affects patient satisfaction and may lead to other complications, such as dehydration, electrolyte imbalance, aspiration pneumonia, increased wound dehiscence, delayed recovery, and prolonged hospital stay. Particularly, in oral and maxillofacial surgeries, PONV may affect the surgical site or the oral cavity [1–3]. Therefore, anesthesiologists should pay special attention to the prevention of PONV in patients undergoing oral and maxillofacial surgery.

In clinical trials, total intravenous anesthesia (TIVA) with propofol has been shown to reduce PONV significantly more than inhalational anesthesia. Therefore, propofol is the recommended anesthetic for patients at high risk of PONV [4, 5]. However, propofol frequently causes injection pain, hypotension, and bradycardia [6–8].

Remimazolam is a novel benzodiazepine that is rapidly metabolized into inactive metabolites via hydrolysis by tissue esterases. The short, context-sensitive half-life of remimazolam makes it possible to use it as a general anesthetic. The efficacy and safety of remimazolam have been compared with those of propofol, and reports indicate some advantages of remimazolam, such as absence of injection pain and a lower incidence of hypotension and bradycardia [9–11].

However, few studies have investigated the incidence of PONV with remimazolam. Therefore, this study was conducted to compare the effects of remimazolam and propofol on PONV in patients undergoing oral and maxillofacial surgery, in which control of PONV is important. The primary outcome was the overall incidence of PONV during the first 24 h after surgery. Secondary outcomes included the severity of nausea, use of rescue antiemetics, severity of postoperative pain, use of rescue analgesia, and quality of recovery.

## Methods

### Study design

This prospective, parallel-group, single-blinded study with balanced randomization (1:1) was approved by the Institutional Review Board (IRB) of Pusan National Dental Hospital (IRB No. PUNDH-2021-10) and registered in the clinical trial registry (KCT0006965). This study was conducted from March 2022 to October 2022 at the Pusan National University Dental Hospital (Yangsan, Republic of Korea). Written informed consent was obtained from all the patients who agreed to participate in this study.

### Participants

The inclusion criteria were adult patients (aged 19–65 years) with American Society of Anesthesiologists (ASA)’s physical status class I-II undergoing oral and maxillofacial surgery. The types of surgery included facial injury and trauma surgery; oral, head, and neck cancer surgery; and oral and maxillofacial surgery (excluding orthognathic surgery). Patients meeting one or more of the following criteria were excluded: hypersensitivity to preoperative benzodiazepines or any component of the drug; acute narrow-angle glaucoma; BMI ≥ 30 kg/m^2^; alcohol or drug dependence; Child-Pugh Class C (decompensated disease in terms of severity of liver disease); history of motion sickness or PONV; and use of antiemetics within 24 h before surgery.

### Randomization

Patients were randomly assigned in a 1:1 ratio to either the remimazolam group (R group) or the propofol group (P group). The randomization was performed by a single investigator using web-based (www.randomization.com) computer-generated random numbers.

The investigators assessing study outcomes and the patients were blinded to group allocation during the study period.

### Anesthesia and perioperative care

No premedication was administered to the patients. All patients were monitored by pulse oximetry, noninvasive monitor of arterial blood pressure, electrocardiography, bispectral index (BIS) monitor, neuromuscular monitor, and carbon dioxide capnography. For the R group, anesthesia was induced with continuous infusion of remimazolam at a rate of 12 mg/kg/hr until loss of consciousness (LoC). Anesthesia was maintained with remimazolam at a rate of 1–2 mg/kg/hr. A target-controlled infusion (TCI) device (Orchestra Base Primea; Fresenius Kabi, France) was used for induction and maintenance of anesthesia with remifentanil (target effect-site concentration: 2.5 ~ 4 ng/ml). For the P group, the same TCI device was used for induction and maintenance of anesthesia with propofol (target effect-site concentration: 3–5 µg/ml) and remifentanil (target effect-site concentration 2.5 ~ 4 ng/ml).

Endotracheal intubation was facilitated with rocuronium 0.6 mg/kg, and rocuronium 0.15 mg/kg was additionally administered if required during surgery. Anesthesia was maintained with a BIS value between 40 and 60. Remimazolam and propofol were reduced if the BIS was less than 40 and increased if the BIS was over 60. The goal was to maintain mean arterial pressure between 60 mmHg and 95 mmHg. Ephedrine 5 mg was administered intravenously if the mean arterial pressure was less than 60 mmHg, and remifentanil was increased if the mean arterial pressure was 95 mmHg or more. All patients received 1000 mg of acetaminophen and 0.3 mg of ramosetron intravenously 20 min before the end of surgery. In the ward, intravenous acetaminophen 1000 mg was administered twice. After completion of surgery, sugammadex 2–4 mg/kg was administered intravenously to reverse the neuromuscular blockade. In group R, flumazenil 0.2 mg was injected intravenously when sufficient spontaneous breathing was restored. Extubation was performed when patients with sufficient spontaneous breathing had a BIS score of 90 or higher, opened their eyes, and followed verbal commands. If consciousness was not restored even after 10 min of administering flumazenil, it was considered delayed emergence, and 0.1 mg of flumazenil was repeatedly administered at 60 s intervals up to a maximum of 1 mg. The patient was extubated and transferred to a post anesthetic care unit (PACU). Patients were monitored for 1 h in the PACU and then transferred to the general ward.

### Data collection

Data collection was performed by investigators who were blinded to group allocation and were not involved in the anesthesia of patients. The clinical characteristics (sex, age, height, weight, and smoking status) of the patients were recorded, and data on the type of surgery, duration of anesthesia, duration of surgery, intraoperative remifentanil, intraoperative fluids, and blood loss were collected. The primary outcome was the overall incidence of postoperative nausea and vomiting during the first 24 h after surgery. Secondary outcomes included the severity of nausea, use of rescue antiemetics, severity of postoperative pain, use of rescue analgesia, and quality of recovery. The intensity of nausea was rated on an 11-point verbal rating scale (VRS). A score of 0 was defined as no nausea, and 10 as severe nausea. The severity of nausea was classified according to the VRS score as follows: no nausea (0), mild (1–3), moderate (4–6), and severe (7–10). Patients who vomited were also included as having severe nausea if the VRS score was 7 or higher. PONV scores were recorded on arrival at the PACU and at 1 h, 6 h, and 24 h postoperatively. All episodes of PONV during the 24 h after surgery were recorded. The rescue antiemetic was ramosetron 0.3 mg. Rescue antiemetics were administered when the patient experienced nausea and requested treatment, when the patient had vomited, or when the VRS score for nausea was ≥ 4. Postoperative pain was assessed using an 11-point numerical rating scale (NRS), where a score of 0 was defined as no pain and a score of 10 as unbearable pain. Ketorolac (30 mg) was administered in cases of severe pain (NRS > 6). If the patient complained of persistent severe pain (NRS > 6) 30 min after ketorolac administration, 1 µg/kg of fentanyl was administered. Quality of recovery was assessed using the Quality of Recovery-40 questionnaire (QoR-40), which was administered 24 h postoperatively. The QoR-40 questionnaire consists of 40 questions, and each question is scored from 1 to 5 for a total of 200 points.

### Sample size calculation

In the unpublished preliminary study, the incidence of PONV was 15% in the P group and 30% in the R group. With a power of 80% and a 5% significance level, 89 patients in each group were required. In consideration of dropout rate, a total of 206 patients were recruited, with 103 patients in each group.

### Statistical analysis

Categorical data are presented as numbers (percentages) and were analyzed using chi-square (χ^2^) or Fisher’s exact test. Continuous data are presented as mean ± standard deviation or medians (interquartile range) and were analyzed using Student’s *t-*test or Mann-Whitney U test. The normality test used skewness and kurtosis, and the normal distribution standard was − 3 ~ + 3 for skewness and − 8 ~ + 8 for kurtosis. A value of p < 0.05 was considered statistically significant. Statistical analyses were performed using SPSS 26.0 (IBM, Armonk, NY, USA).

## Results

**Fig. 1:**
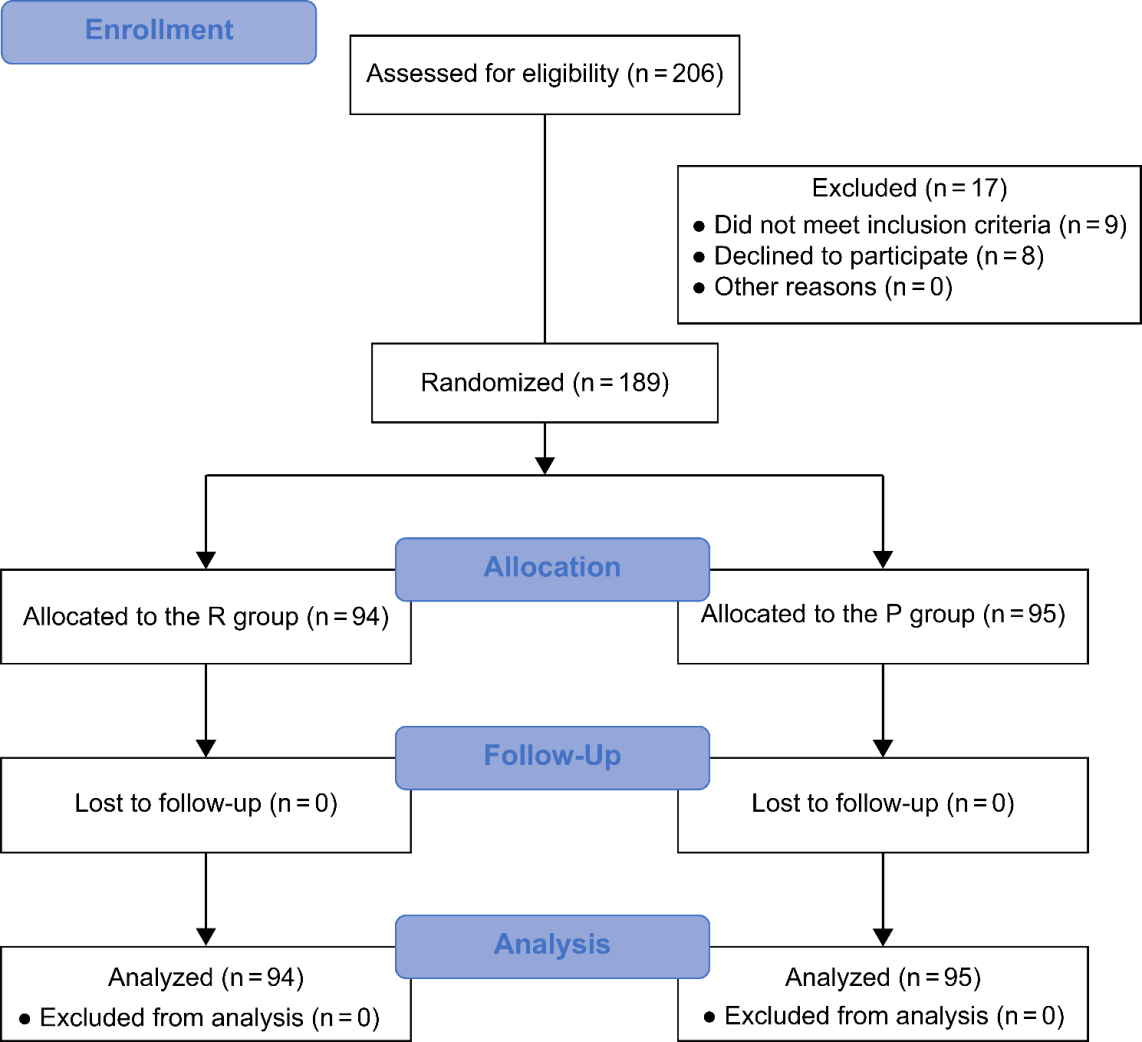
CONSORT diagram

A total of 206 patients were included in this study during the period between March 2022 and October 2022. Nine patients did not meet the inclusion criteria, and eight patients did not provide consent to participate in the study and were excluded. Therefore, 189 patients, [(R group, n = 94); (P group, n = 95)] completed the study (Fig. 1).

**Table 1:** Clinical characteristics and intraoperative variables of patients

	R group (n = 94)	P group (n = 95)
Age (years)	41.7 ± 12.2	43.3 ± 13.2
Height (cm)	168.7 ± 7.4	168.4 ± 8.3
Weight (kg)	68.3 ± 13.1	67.4 ± 11.6
BMI (kg/m^2^)	23.8 ± 3.3	23.7 ± 2.9
ASA (I/II)		
I	55 (58.5%)	54 (56.8%)
II	39 (41.5%)	41 (43.2%)
Gender		
Female	33 (35.1%)	36 (37.9%)
Male	61 (64.9%)	59 (62.1%)
Smoking (No/Yes)		
No	69 (73.4%)	72 (75.8%)
Yes	25 (26.6%)	23 (24.2%)
Type of surgery		
Cyst enucleation	50 (53.2%)	53 (55.8%)
Tooth extraction	21 (22.3%)	22 (23.2%)
Tumor resection	23 (24.5%)	20 (21%)
Duration of surgery (min)	42.8 ± 24.5	46.4 ± 22.9
Duration of anesthesia (min)	71.8 ± 26.1	75.0 ± 22.4
Intraoperative fluids (ml)	307.4 ± 127.0	330.0 ± 116.6
Intraoperative remifentanil (µg)	705.3 ± 254.0	634.7 ± 321.0
Blood loss (ml)		
≤ 100	91 (96.8%)	91 (95.8%)
101–499	3 (3.2%)	4 (4.2%)

Data are presented as number of patients (percentages) or mean ± standard deviation. ASA American Society of Anesthesiologists, BMI body mass index

Clinical characteristics of the patients, PONV risk factors (female sex and nonsmoking status), and intraoperative variables related to anesthesia and surgery were similar between the two groups (Table 1).

**Table 2:** Incidence of PONV and antiemetic treatment during the first 24 h after surgery

	R group (n = 94)	P group (n = 95)	P-value
0–1 h			
Nausea	6 (6.4%)	4 (4.2%)	0.536
Vomiting	1 (1.1%)	0 (0%)	0.497
PONV	6 (6.4%)	4 (4.2%)	0.536
Rescue antiemetics	6 (6.4%)	4 (4.2%)	0.536
1–6 h			
Nausea	6 (6.4%)	6 (6.3%)	0.985
Vomiting	1 (1.1%)	1 (1.1%)	1.000
PONV	6 (6.4%)	6 (6.3%)	0.985
Rescue antiemetics	4 (4.3%)	5 (5.3%)	1.000
6–24 h			
Nausea	1 (1.1%)	1 (1.1%)	1.000
Vomiting	0 (0%)	0 (0%)	-
PONV	1 (1.1%)	1 (1.1%)	1.000
Rescue antiemetics	0 (0%)	1 (1.1%)	1.000
0–24 h			
Nausea	11 (11.7%)	10 (10.5%)	0.821
Vomiting	2 (2.1%)	1 (1.1%)	0.621
PONV	11 (11.7%)	10 (10.5%)	0.797
Rescue antiemetics	10 (10.6%)	10 (10.5%)	0.980

Data are presented as number of patients (percentage). PONV postoperative nausea and vomiting

During the first 24 h after surgery, the incidence of PONV was 11.7% in the R group and 10.5% in the P group, and there were no significant differences between the two groups at 0–1 h, 1–6 h, 6–24 h, or during the entire 24 h (P > 0.05). The incidence of nausea for the entire 24 h was 11.7% in the R group and 10.5% in the P group, and the incidence of vomiting for the first 24 h was 2.1% in the R group and 1.1% in the P group (P > 0.05). There was no statistically significant difference in the incidence of nausea and vomiting between the two groups at 0–1 h, 1–6 h, 6–24 h, and 24 h postoperatively (P > 0.05). We also found that 10.6% of patients in the R group and 10.5% of patients in the P group required rescue antiemetics within 24 h of surgery (P > 0.05), and there was no difference between the two groups in terms of the use of antiemetics for 0–1 h, 1–6 h, 6–24 h, and for the entire 24 h. (P > 0.05) (Table 2).

The severity of nausea during 24 h postoperatively (Table 3), the severity of postoperative pain, and the use of rescue analgesia (Table 4) were not significantly different between the two groups (P > 0.05). None of the patients received rescue analgesia more than once or required fentanyl for rescue analgesia.

**Table 3:** Severity of postoperative nausea

	R group (n = 94)	P group (n = 95)	P-value
0–1 h			
Mild	0 (0%)	0 (0%)	0.771
Moderate	4 (4.3%)	3 (3.2%)	
Severe	2 (2.1%)	1 (1.1%)	
1–6 h			
Mild	2 (2.1%)	1 (1.1%)	0.924
Moderate	3 (3.2%)	4 (4.2%)	
Severe	1 (1.1%)	1 (1.1%)	
6–24 h			
Mild	1 (1.1%)	0 (0%)	0.368
Moderate	0 (0%)	1 (1.1%)	
Severe	0 (0%)	0 (0%)	

Data are presented as number of patients (percentage).

**Table 4:** Severity of postoperative pain and analgesic treatment

	R group (n = 94)	P group (n = 95)	P-value
Pain score (NRS)			
0–1 h	4.0 (4.0 to 4.0)	4.0 (4.0 to 5.0)	0.245
1–6 h	3.0 (3.0 to 3.0)	3.0 (3.0 to 3.0)	0.779
6–24 h	2.0 (1.0 to 2.0)	2.0 (1.0 to 2.0)	0.146
Rescue analgesia			
0–1 h	8 (8.5%)	9 (9.5%)	0.817
1–6 h	1 (1.1%)	0 (0%)	0.497
6–24 h	3 (3.2%)	4 (4.2%)	1.000

Data are presented as number of patients (percentages) or median (interquartile range). NRS numerical rating scale.

There was no statistically significant difference between the two groups in the total QoR-40 score (p > 0.05). None of the five dimensions differed between the groups (P > 0.05) (Table 5).

**Table 5:** Postoperative QoR-40 scores at 24 h

Postoperative QoR-40	R group (n = 94)	P group (n = 95)	P-value
Total QoR-40	176.7 ± 12.5	177.1 ± 13.4	0.836
Physical comfort	54.0 ± 3.3	54.0 ± 3.7	0.967
Emotional state	40.0 (39.0 to 42.0)	41.0 (38.0 to 42.0)	0.621
Psychological support	31.4 ± 3.1	31.5 ± 3.0	0.694
Physical independence	20.4 ± 3.9	20.2 ± 4.4	0.776
Pain	31.0 ± 2.7	31.3 ± 3.5	0.628

Data are presented as mean ± standard deviation or median (interquartile range). QoR-40 Quality of Recovery-40 questionnaire.

## Discussion

Although remimazolam is a novel drug, several studies have reported its use as an anesthetic. Its sedative effect can be rapidly reversed by flumazenil. Therefore, it is used successfully in clinical practice [9–12]. One study reported that the incidence of PONV was lower with remimazolam than with inhalational anesthetics [13]. However, there have been few studies on the PONV associated with remimazolam and propofol in TIVA. In this study, the effects of remimazolam and propofol on PONV were compared by using TIVA. The main finding of this study is that the use of remimazolam did not increase the incidence of PONV compared to the use of propofol, which is already known to have antiemetic effects, in patients undergoing oral and maxillofacial surgery. In addition, there were no differences between the two groups in terms of the severity of nausea, use of rescue antiemetics, severity of postoperative pain, use of rescue analgesia, and quality of recovery.

Previous studies have reported that remimazolam does not increase PONV compared with propofol. Choi et al. [14] compared the quality of recovery between remimazolam and propofol in patients undergoing open thyroidectomy with TIVA. Guo et al. [15] compared the sedative effects of remimazolam and propofol in elderly patients undergoing gastrointestinal endoscopy, and found no difference in PONV between the two groups. Similarly, in the present study, there was no difference in the incidence of PONV between the two groups.

Prakash et al. [16] reported that midazolam may reduce the incidence of PONV by inhibiting the chemoreceptor trigger zone activity and reducing the release of 5-hydroxy tryptamine (5-HT) by binding to the gamma-aminobutyric acid (GABA) receptor. However, since remimazolam is rapidly metabolized by carboxylesterase, the context-sensitive half-time (CSHT) is 7–8 min for a 2-hour continuous infusion, which is similar to that of propofol and much shorter than that of midazolam [17]. In addition, administration of flumazenil, a benzodiazepine receptor antagonist, to all patients in group R in this study may have reduced the effect of residual remimazolam. Thus, it is not possible to determine whether the low incidence of PONV in Group R is due to the effect of remimazolam or elimination of remimazolam. Therefore, further studies are needed to determine whether remimazolam has a prophylactic effect on PONV.

Postoperative pain is considered a risk factor for PONV [18, 19]. Therefore, intravenous acetaminophen was administered as a prophylactic measure before the completion of surgery and on the day of the surgery. As a result, most of the patients did not require rescue analgesia. Moreover, pain severity did not differ between the groups. Therefore, the effect of postoperative pain on PONV was not significant.

There were no differences between groups in the QoR-40 used to compare the quality of recovery. Considering that PONV has a significant impact on recovery, the similar incidence of PONV between the two groups may have contributed to this outcome [20].

Despite various efforts to reduce PONV, the incidence of PONV is still 20–30%, and can be as high as 80% in high-risk groups [21]. In this study, the incidence of PONV within 24 h after surgery was 11.7% in the R group and 10.5% in the P group. The incidence of PONV was lower than that reported in the existing literature, and this may be attributed to differences in the study population and anesthetic practice.

The Apfel simplified risk score is a tool commonly used for PONV risk assessment, and it has been used to identify female sex, nonsmoking status, history of PONV or motion sickness, and postoperative opioid use as risk factors for PONV occurrence [22]. Since a history of PONV or motion sickness is considered a risk factor, and patients meeting this condition were excluded from the study, this may have also contributed to the low incidence of PONV among patients enrolled in the present study.

Antiemetics that are used for the relief of PONV act by blocking the dopamine (D_2_), histaminic (H_1_), 5-hydroxytryptamine_3_ (serotonin), and muscarinic-cholinergic receptors in the chemoreceptor trigger zone [23]. All patients in the present study were administered ramosetron, a 5-HT_3_ receptor antagonist, before the end of surgery. Ramosetron has a longer plasma half-life (5.8 ± 1.2 h) and duration of action than other 5-HT_3_ receptor antagonists [24]. The use of prophylactic antiemetics is thought to have also contributed to reducing the incidence of PONV.

Furthermore, sugammadex was administered as a reverse non-depolarizing muscle relaxant, which may have reduced the incidence of PONV. Cholinesterase inhibitors are used to reverse non-depolarizing neuromuscular blockade, which may be associated with increased incidence of PONV. Several reports have stated that the reversal of neuromuscular blocking action using sugammadex induces less PONV than the use of cholinesterase inhibitors such as neostigmine [25, 26].

This study has several limitations. First, we calculated the sample size based on an unpublished preliminary study. Second, since the analysis did not involve categorization according to the type of surgery, the postoperative oral conditions were not consistent among the patients. Third, PONV beyond 24 h after surgery was not examined. Therefore, further studies with a sufficient number of participants undergoing the same type of surgery are needed to investigate the incidence of PONV beyond this point. In addition, we did not compare the two groups for many factors that affect the occurrence of PONV, such as hypotension and hypercarbia [27, 28]. Further, due to the different characteristics of the two anesthetics, double blinding was not possible for the anesthesiologist involved in anesthesia. Finally, the use of flumazenil in the remimazolam group is a limitation as it introduces a potential confounding variable to the two balanced groups.

## Conclusions

In conclusion, for noninvasive oral and maxillofacial surgery, remimazolam did not increase the incidence of PONV during the first 24 h post-surgery compared to what was observed with the use of propofol, and there were no significant differences in terms of nausea severity. Therefore, remimazolam can be considered as a useful alternative to propofol in TIVA.

## Data Availability

The data in this study are available upon request from the corresponding author.

## Abbreviations

ASA: American Society of Anesthesiologists
PACU: Post anesthetic care unit
PONV: Postoperative nausea and vomiting
TIVA: Total intravenous anesthesia.
